# Vagus nerve stimulation modulates hippocampal inflammation caused by continuous stress in rats

**DOI:** 10.1186/s12974-022-02396-z

**Published:** 2022-02-02

**Authors:** Uk Namgung, Ki-Joong Kim, Byung-Gon Jo, Jong Min Park

**Affiliations:** Department of Oriental Medicine, Institute of Bioscience and Integrative Medicine, Daejeon University, Daehak-ro 62, Daejeon, 34520 South Korea

**Keywords:** Continuous stress, Vagus nerve stimulation, Inflammatory cytokines, Neuroinflammation, Hippocampus, Cholinergic anti-inflammation

## Abstract

**Background:**

Previous studies have shown that vagus nerve stimulation (VNS) can attenuate inflammatory responses in peripheral tissues and also improve some neurological disorders and cognitive function in the brain. However, it is not clear how VNS is involved in neuropathological processes in brain tissues. Here, we investigated the regulatory effects of VNS on the production of proinflammatory cytokines in the hippocampus of an animal model of continuous stress (CS).

**Methods:**

CS was induced by placing rats in cages immersed with water, and acute or chronic electrical stimulation was applied to the cervical vagus nerve of CS animals. Protein levels in the gastric and hippocampal tissues were measured by western blotting and protein signals analyzed by immunofluorescence staining. von Frey test and forced swimming test were performed to assess pain sensitivity and depressive-like behavior in rats, respectively.

**Results:**

Levels of TNF-α, IL-1β, and IL-6 in the gastric and hippocampal tissues were significantly increased in CS animals compared to the untreated control and downregulated by acute VNS (aVNS). Iba-1-labeled microglial cells in the hippocampus of CS animals revealed morphological features of activated inflammatory cells and then changed to a normal shape by VNS. VNS elevated hippocampal expression of α7 nicotinic acetylcholine receptors (α7 nAChR) in CS animals, and pharmacological blockade of α7 nAChR increased the production of TNF-α, IL-1β, and IL-6, thus suppressing cholinergic anti-inflammatory activity that was mediated by VNS. Chronic VNS (cVNS) down-regulated the hippocampal production of active form of caspase 3 and 5-HT1A receptors and also decreased levels of TNF-α, IL-1β, and IL-6 in the gastric and hippocampal tissues of CS animals. Pain sensitivity and depressive-like behavior, which were increased by CS, were improved by cVNS.

**Conclusions:**

Our data suggest that VNS may be involved in modulating pathophysiological processes caused by CS in the brain.

## Background

Continuous stress (CS) can be induced in experimental animals by maintaining them in water-immersed cages for several days, and activation of microglial cells in the spinal cord of CS animals was shown to be related to chronic pain such as hyperalgesia and mechanical allodynia [1–3]. Studies further showed that CS alters endocrine function (e.g., downregulation of growth hormone production from atrophied somatotrophs in the pituitary gland and increased production of α-melanocyte stimulating hormone in the intermediate lobe of the pituitary gland) [4, 5]. Having noted that the symptoms observed from CS animals displayed the similarity partially with those of the patients suffering from myalgic encephalomyelitis/chronic fatigues syndromes (ME/CFS), CS animal was proposed as a model of ME/CFS [1, 2].

Previous studies have provided convincing evidence that vagus nerve activity is involved in regulating inflammation and several diseases in visceral organs such as chronic bowel disease, irritable bowel syndrome, obesity, diabetes and others [6, 7]. Here, acetylcholine neurotransmitters released from the efferent fibers of the vagus nerve interact with ɑ7 nicotinic acetylcholine receptors (ɑ7 nAChR) in target cells (e.g., splenic macrophages) and subsequently induce the JAK2 activation of STAT3 and NF-kB leading to the inhibition of the expression of proinflammatory cytokine genes [8, 9]. Since VNS is given to the exposed nerve in experimental animals, anti-inflammatory effects can also be influenced by afferent nerve activity through feedback loop in the dorsal vagal complex [10–12]. Moreover, VNS acting on the ascending fibers that transmit signals to the forebrain was reported to enhance memory formation and improve mental illnesses such as epilepsy and depression [13–15]. Cell-specific transneuronal labeling has identified the neural circuits connecting the guts and forebrain that activate the reward system, thus demonstrating the vagus nerve activity as a modulator of brain–gut axis [16]. Ascending vagus nerve activity may be involved in activating brainstem nuclei for neuromodulation and affect the limbic system and cerebral cortex, modulating emotional and cognitive function in the brain [7]. We have recently reported that the afferent component of VNS increased cholinergic nerve activity in the dorsal vagal complex and resulted in the upregulation of α7 nAChR levels in the liver [12]. α7 nAChR activity in the brain was shown to participate in the regulation of NMDA and GABA receptor activation and correlate with the occurrence of Schizophrenia [17–19]. There is a report showing microglial activation of α7 nAChR receptors which is related to the suppression of neuroinflammation [20]. However, direct evidence verifying VNS-modulated neuroinflammation in the brain via α7 nAChR has not been reported so far.

Continuous stress deteriorates mental and physical activities in experimental animals and may cause a broad spectrum of neuroimmunopsychological abnormalities. As an initial step of exploring the potential effects of VNS on the regulation of neuroimmune disorders such as CFS, we investigated VNS-mediated changes of pathophysiological and behavioral responses in the hippocampus of CS animals. The present study provides evidence that VNS plays a role in regulating hippocampal production of inflammatory cytokines and behavioral abnormalities associated with CS.

## Materials and methods

### Animals

Sprague–Dawley rats (male and female, 7–8 weeks, 200–250 g) were purchased (Samtako Inc., Seoul, Korea) and acclimatized for 7 days before the use for the experiments in ventilated animal room with 22–23 °C of temperature and 60% humidity under a standard 12 h light and 12 h dark cycle (lights on from 7:00 am to 7:00 pm). All animals were freely accessed to commercial chow (Samyang Co., Eumseong, Korea) and drink water. In this study, a total of 111 rats were used and their use in the present study categorized into the acute VNS (aVNS) and the chronic VNS (cVNS) experiments. As for aVNS experiments, 51 rats were randomly assigned with an equal number into CS plus sham (CS+Sham), CS plus aVNS (CS+aVNS) and untreated control (CTL) groups. As for cVNS experiments, a total of 60 rats were assigned into CS+Sham, CS+cVNS, and CTL groups, distributing 20 animals equally to each experimental group. All animals were survived during and after the surgery, and thus, no animal was excluded for data analysis. All protocols involving live and postoperative animal care were approved by the Daejeon University Institutional Animal Use and Care Committee and were in accordance with the Animal‐Use Statement and Ethics Committee Approval Statement for Animal Experiments provided by Daejeon University (Protocol number: DJUARB2019-029, Daejeon, Korea).

### CS animal model

An experimental procedure for CS in rats was essentially the same as described previously [2]. Briefly, rats were randomly designated to CS or untreated control group (CTL). For the performance of CS, each rat was placed in a single cage filled with 23 °C of water with a height of 1.5 cm and was transferred every 24 h to a new cage filled with the same amount of water. Animals were given CS for 5 consecutive days during which food and water were provided. Twenty four hours after the final CS, animals were subjected to VNS or sham surgery, as described below.

### Acute and chronic vagus nerve stimulation

After the CS, rats were anesthetized with ketamine (80 mg/kg; Yuhan, Seoul, Korea) and xylazine (5 mg/kg; Bayer, Leverkusen, Germany). VNS in rats was given as described in our previous study [12, 21]. Briefly, the anterior surface of the neck and the muscles were incised and the larynx was lifted. The lower muscle of the larynx was cut to expose the right cervical vagus nerve and carotid artery, and the nerve was carefully separated from the carotid artery. For aVNS, the exposed vagus nerve was placed into a U-shape of a bipolar electrode of tungsten wire (250 μm diameter, A-M System Inc., Sequim, WA, USA). The electrical current (10 mA, 5 Hz, 5 ms of pulse duration) was applied for 5 min using the electrical stimulator (Isolated Pulse Stimulator Model 2100, A-M Systems Inc.). For cVNS, rats were implanted with a microelectrode cuff (Inner diameter 0.75 mm; Microprobes, Gaithersburg, MD, USA) as described previously [21] and, after the recovery from anesthesia, returned to an animal room. The first electrical stimulation was given 24 h after the implantation of the microelectrode with the same stimulation condition as the acute stimulation, and the same intensities of stimulation were applied on a daily basis for 7 consecutive days. cVNS was applied to individual animals under the awake state. For the sham treatments, CS animals were anesthetized by ketamine and xylazine, the right vagus nerves were exposed, and the skins were suture. All experimental groups of animals underwent the same procedures of recovery and maintenance and were sacrificed with an overdose of ketamine (150 mg/kg, i.p.).

### Intracerebroventricular injection of drug

Rats were anesthetized with ketamine and xylazine with the same dose as above and placed in the stereotaxic apparatus for drug injection. The skull of the forebrain was perforated using a drill. An α7 nAChR antagonist methyllycaconitine citrate salt (MLA; 1 μg/μl in 0.9% NaCl; Sigma-Aldrich, St. Louis, MO, USA) or an equivalent volume of saline solution was injected into the lateral ventricle (coordinate AP: 0.8 mm; L: 1.5 mm; DV: 3.5 mm) [22] using a micropump (Pump 11 Elite, Harvard Apparatus) with a flow rate of 5 μl/min for 2 min [23] and the injection needle remained penetrated for 3 min to prevent drug reflux and also allow drug to diffuse into the surrounding area.

### Western blot analysis

The hippocampal tissue was dissected from rats and sonicated in RIPA buffer (150 mM NaCl, 1.0% CA-630, 0.5% sodium deoxycholate, 0.1% SDS, 50 mM Tris, pH 8.0; Thermo Fisher Scientific, MA, USA) supplemented with protease inhibitor and phosphatase inhibitor cocktails (Roche Diagnostics, Canton, Switzerland). The lysate was centrifuged at 12,000 rpm for 15 min at 4 °C and the supernatant was collected. Protein (20 μg) was resolved by SDS polyacrylamide gel electrophoresis and transferred to a PVDF membrane. The membrane was blocked with 5% BSA in 1X TBST (0.1% tween 20 in Tris-buffered saline) and incubated with the primary antibody followed by reaction with secondary antibody. Immunoblotting was performed using primary antibodies including anti-TNF-α (Rabbit-polyclonal, 1:2000; Abcam, Cambridge, UK), anti-IL-1β (Rabbit-polyclonal, 1:2000; Abcam), anti-IL-6 (Mouse-monoclonal, 1:2000; Abcam), anti-α7 nicotinic acetylcholine receptor (Rabbit-polyclonal, 1:200, Alomone Labs, Jerusalem, Israel), anti-choline acetyltransferase (Rabbit-monoclonal, 1:2000; Abcam), anti-5HT1A receptor (Rabbit-polyclonal, 1:1000, Abcam), anti-Iba-1 (Mouse-monoclonal, 1:1000; Fujifilm, Minato, Japan), anti-cleaved Caspase-3 (Rabbit-polyclonal, 1:1000; Cell Signaling Technology, Beverly, MA, USA) and anti-β-actin (Mouse-monoclonal, 1:50,000; Sigma-Aldrich) antibodies. Secondary antibodies were anti-rabbit HRP (1:5000; Cell Signaling Technology) and anti-mouse HRP (1:5000; Cell Signaling Technology) antibodies. The intensity of each protein band was analyzed using the i-Solution software (version 21.1, Image & Microscope Technology, Daejeon, Korea) and compared with that of β-actin protein band.

### Reverse transcription polymerase chain reaction

Total RNA was extracted from the hippocampus using trizol reagent (Thermo Fisher Scientific). cDNA was synthesized by incubating isolated RNA in the reaction containing 50 mM Tris–HCl, 75 mM KCl, 3 mM MgCl_2_, 10 mM DTT, 104 μM dNTP, RNasin (30 U), random primers (16 μM, Promega, Madison, Wisconsin, USA), and MMLV reverse transcriptase (200 U, Promega, Wisconsin, USA) for 2 h at 37 °C. The primer sequences for RT-PCR were the forward primer (5′-CCTGCTCCCCAACACATGAT-3′) and the reverse primer (5′-GACATGAGGATGCCGATGGT-3′) for α7 nAChR mRNA and the forward primer (5'-CACACTGTGCCCATCTATGA-3′) and the reverse primer (5′-CCATCTCTTGCTCGAAGTCT-3′) for actin mRNA.

### Gastric tissue histology

Animals were deprived of food pellets for 24 h before sacrifice. The stomachs were dissected, washed with 1× PBS, and filled with 1× PBS. After freezing at − 20 °C for 10 min, the stomachs were cut from the pylorus to the cardiac orifice, and the gastric mucosal lesion was evaluated by spreading the cut stomach and digitally photographed (Nikon E-600, Tokyo, Japan). For hemotoxylin and eosin staining, rats were transcardially perfused with 4% paraformaldehyde in 1× PBS, and the gastric tissue was isolated and postfixed for 2 h with 4% paraformaldehyde and subsequently immersed with 30% sucrose solution at 4 °C for 2 days. Then, the tissue was rapidly frozen by dipping into − 80 °C of 2-methylbutane (Sigma-Aldrich) and the transverse sections (5 μm thickness) were prepared using a cryostat (Leica CM1850, Wetzlar, Germany) and mounted on the superfrost plus microscope slide (Thermo Scientific, Waltham, MA, USA). Slides were dipped into hematoxylin solution (HEMH-OT-1L, BioGnost, Zagreb, Croatia) for 40 s and washed with distilled water. Slides were also stained with Eosin Y solution (EOYA-05-OT-1L, BioGnost) for 10 min and dehydrated gradually through 50%, 60%, 75%, 90%, and 100% ethanol for 5 min each. The sections were cleared with xylene (Sigma-Aldrich) for 5 min and mounted with xylene-based mounting medium.

### Immunofluorescence staining

Rats were anesthetized with an overdose of ketamine and xylazine and perfused with 1X phosphate buffered saline (PBS) and 4% paraformaldehyde in PBS. The whole brain was dissected and immersed overnight in 30% sucrose in PBS solution. After rapid freezing at − 80 °C with 2-methylbutane (Sigma-Aldrich), tissues were cut using a cryostat (Leica CM1850, Wetzlar, Germany) and thaw-mounted on the slide (16 µm thickness). Sections were fixed, permeabilized, treated with blocking solution (2.5% BSA, 2.5% horse serum, 0.1% Triton X-100 in 1× PBS), and incubated with primary antibodies for 24 h at 4 °C, washed three times with 1× PBST and incubated with secondary antibodies for 2 h at room temperature in a dark room. The primary antibodies used were anti-IL-1β (Rabbit-polyclonal, 1:2000; Abcam), anti-5HT1AR (Rabbit-polyclonal, 1:200; Abcam), anti-Iba-1 (Rabbit-polyclonal, 1:1000; Fujifilm), anti-cleaved caspase 3 (Rabbit-polyclonal, 1:1000; Cell Signaling Technology), anti-α7 nAChR (Rabbit-polyclonal, 1:200; Alomone Labs) and anti-NeuN (Mouse-monoclonal, 1:1000; BD Biosciences, Franklin Lakes, NJ, USA) antibodies. Rhodamine-goat anti-rabbit IgG (1:400, Molecular Probes, Eugene, OR, USA) and fluorescein-goat anti-mouse IgG (1:400, Molecular Probe) were used as secondary antibodies. For nuclear staining, sections were incubated with 2.5 μg/ml of Hoechst 33258 (bis-benzimide, Sigma-Aldrich) for 10 min before the final washing with 1× PBST. Branch lengths of microglial cells were quantified by i-Solutions of software program (Image & Microscope). The distances between the surface of cell body and the tip of each branch were measured and averaged per each microglial cell. In each experimental group, randomly selected 15–20 cells per animal were analyzed and the data were averaged from four independent animal preparations. Then, the statistical comparisons were made among experimental groups.

### Behavioral tests

For pain sensitivity test, rats were placed in the metal mesh cuboid-shaped acrylic plastic chambers and allowed to adapt to the environment for 30 min before the test. The filament was applied to force the mid-plantar of the hind paw for intervals of 5 s. The von Frey threshold in *g* values was determined by the observation of withdrawal response to filament force (Almemo 2450, Ahlborn equipment Inc., Sayner, WI, USA) and the value per animal was determined by averaging 20 or 40 different measures. For the forced swimming test, each rat was examined in a transparent cylindrical chamber (height 50 cm, diameter 25 cm) filled with water at 27 °C (depth 35 cm). Twenty-four hours before the test, animals were adjusted in water for 15 min. Following 1 min adjustment in water, animals were placed in a water chamber for 5 min and their swimming behaviors were monitored and analyzed using a Smart version 2.5 video tracking system (Panlab, Barcelona, Spain). Immobility scores were determined by measuring the time periods that animals kept maintaining the head above the water without any additional actions, and swimming scores were determined by measuring the time period that the animals were moving actively above the water.

### Statistical analysis

Data were analyzed as mean ± standard deviation (SD). The mean values of the data in individual groups were compared using one-way ANOVA and Tukey’s test for multiple comparisons or two-way ANOVA repeated measures with Sidak multiple comparison test (GraphPad Prism 7.00). Statistically significant differences were set at *p < 0.05, **p < 0.01, ***p < 0.001.

## Results

### Gastric inflammation in CS animals is ameliorated by aVNS

**Fig. 1:**
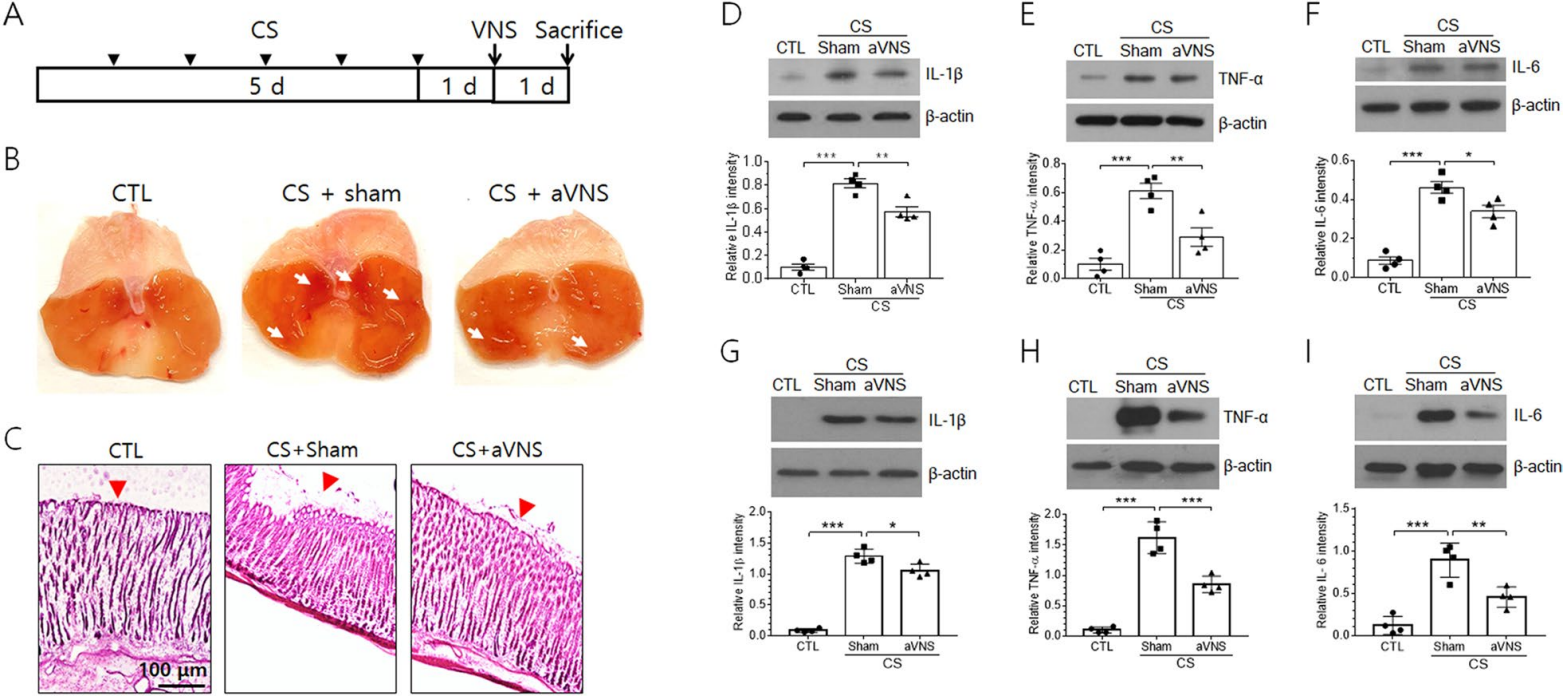
Regulation of gastric mucosal lesion and production of inflammatory cytokines in CS animals by aVNS. **A** Scheme showing the time course of animal treatments before the preparation of gastric tissues. **B** Representative photographic images of the inner surface of gastric mucosal tissues. **C** Microphotographs of hemotoxylin and eosin stained gastric tissues. The surface of mucosa layer in the stomach is marked by arrowheads. **D**–**I** Western blot analysis of IL-1β, TNF-α, and IL-6 proteins from the lysates of gastric tissues in male rats (**D**–**F**) and in female rats (**G**–**I**). Representative images of four independent experiments are shown in upper panels, and quantification of the band intensity for individual proteins are plotted in lower panels. In **D**–**I**, *p < 0.05, **p < 0.01, ***p < 0.001 (One-way ANOVA)

It was previously reported that CS in experimental animals causes severe inflammation in the stomach [24]. Here, we investigated the pathological responses of gastric tissues in male rats given CS for 5 days and further examined the effects of VNS on gastric inflammation (Fig. 1A). The gastric lumen of CS animals was swollen and showed mucosal membrane lesion such as erosion and ulcerations accompanied by erythermatous gastric mucosa, compared to the control animal, and the lesion was ameliorated by VNS (marked by white arrows in Fig. 1B). Hemotoxylin and eosin staining of transverse sections revealed traces of hemorrhage and tissue disruption in the mucosal membrane of CS animals (marked red arrowheads in Fig. 1C) and tissue morphology was largely improved by aVNS. Gastric levels of proinflammatory cytokines IL-1β, TNF-α, and IL-6 were greatly elevated in CS animals, and the levels were significantly reduced by aVNS (Fig. 1D–F). Considering the possible difference in inflammatory responses between male and female rats given CS, a model of diseases such as CFS affecting more women than men [25], we also examined the effects of aVNS on the production of inflammatory cytokines in female rats. As shown in Fig. 1G–I, levels of IL-1β, TNF-α, and IL-6 in the gastric tissue were significantly increased by CS and decreased by aVNS, suggesting similar anti-inflammatory effects of VNS in both male and female animals.

### Production of inflammatory cytokines and microglial activation in the hippocampus of CS animals are alleviated by aVNS

**Fig. 2:**
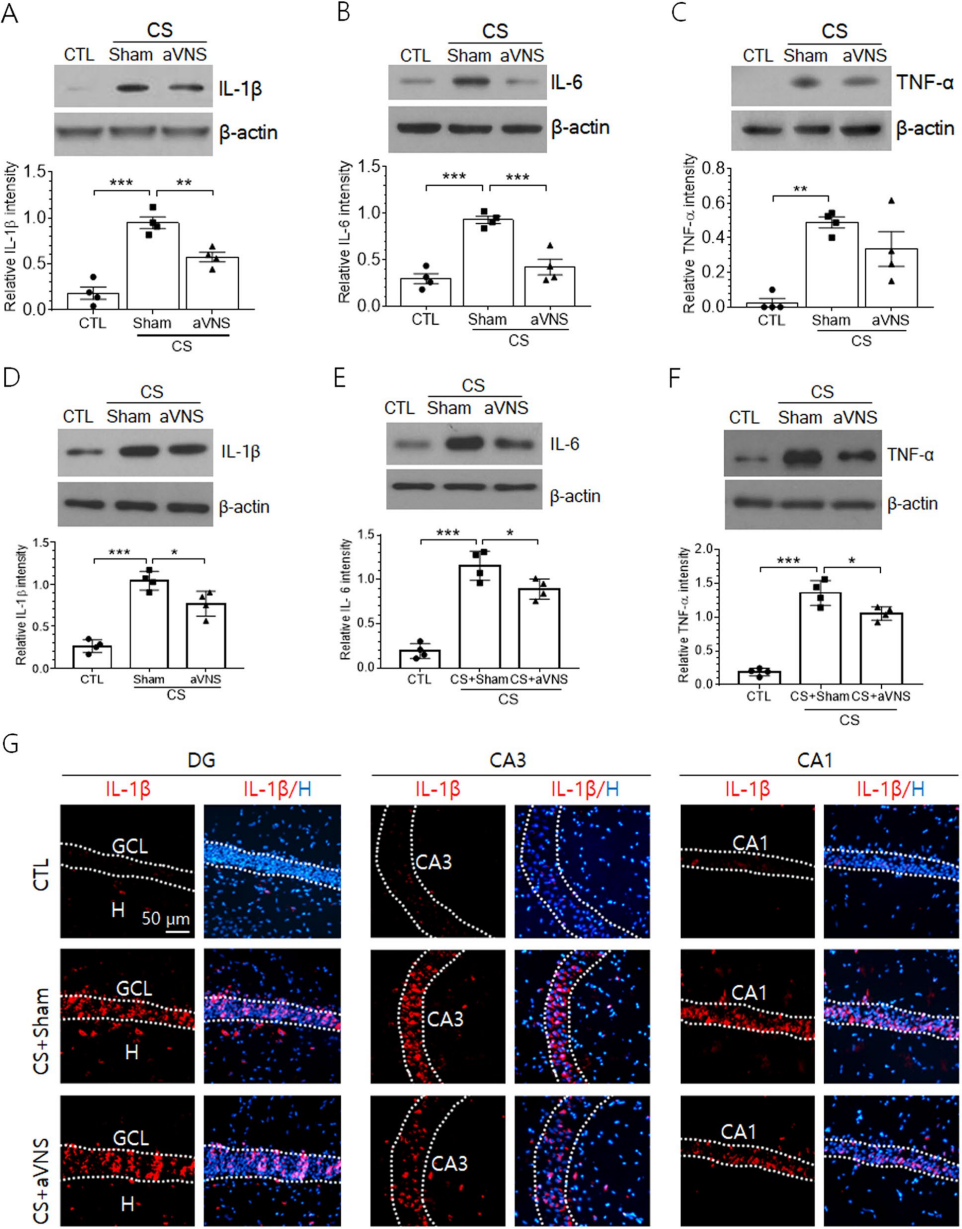
Hippocampal induction of inflammatory cytokines IL-1β, IL-6, and TNF-α in CS animals is downregulated by aVNS. Western blot analysis of hippocampal IL-1β (**A**), IL-6 (**B**) and TNF-α (**C**) proteins in male rats and of the same proteins in female rats (**D**–**F**). Images in (**A**–**F**) are the representatives from four independent experiments (upper panels). Quantification of band intensity of each protein relative to actin is plotted (lower panels). *p < 0.05, **p < 0.01, ***p < 0.001 (One-way ANOVA). **G** Immunofluorescence images of IL-1β signals (red) in the granule cell layer (GCL) of the dentate gyrus (DG), CA3 and CA1 pyramidal cell layers. The nuclei of neurons were visualized by staining with Hoechst 33258 (blue)

We set out the experiment examining the effects of VNS on pathological responses in the hippocampus of CS animals. The levels of IL-1β, IL-6, and TNF-α in male rats were very low in the hippocampus of the control group and strongly induced by CS (Fig. 2A–C). Then, the application of aVNS significantly reduced IL-1β and IL-6 protein levels. Mean level of TNF-α was decreased by 31% after VNS but failed to show statistical significance (*p* = 0.23, One-way ANOVA). Examination of hippocampal IL-1β, IL-6, and TNF-α levels in female rats showed similar increases after CS and significant reduction by aVNS (Fig. 2D–F). We also investigated the distribution of inflammatory cytokine signals in the hippocampal subfields by immunofluorescence staining. IL-1β signals were clearly induced after CS in hippocampal neurons in the granule cell layer (GCL) and CA3 and CA1 pyramidal cell layers while showing much weak signals in non-neuronal hippocampal areas (Fig. 2G). IL-1β signals in CA3 and CA1 pyramidal cell layers were notably attenuated by VNS.

**Fig. 3:**
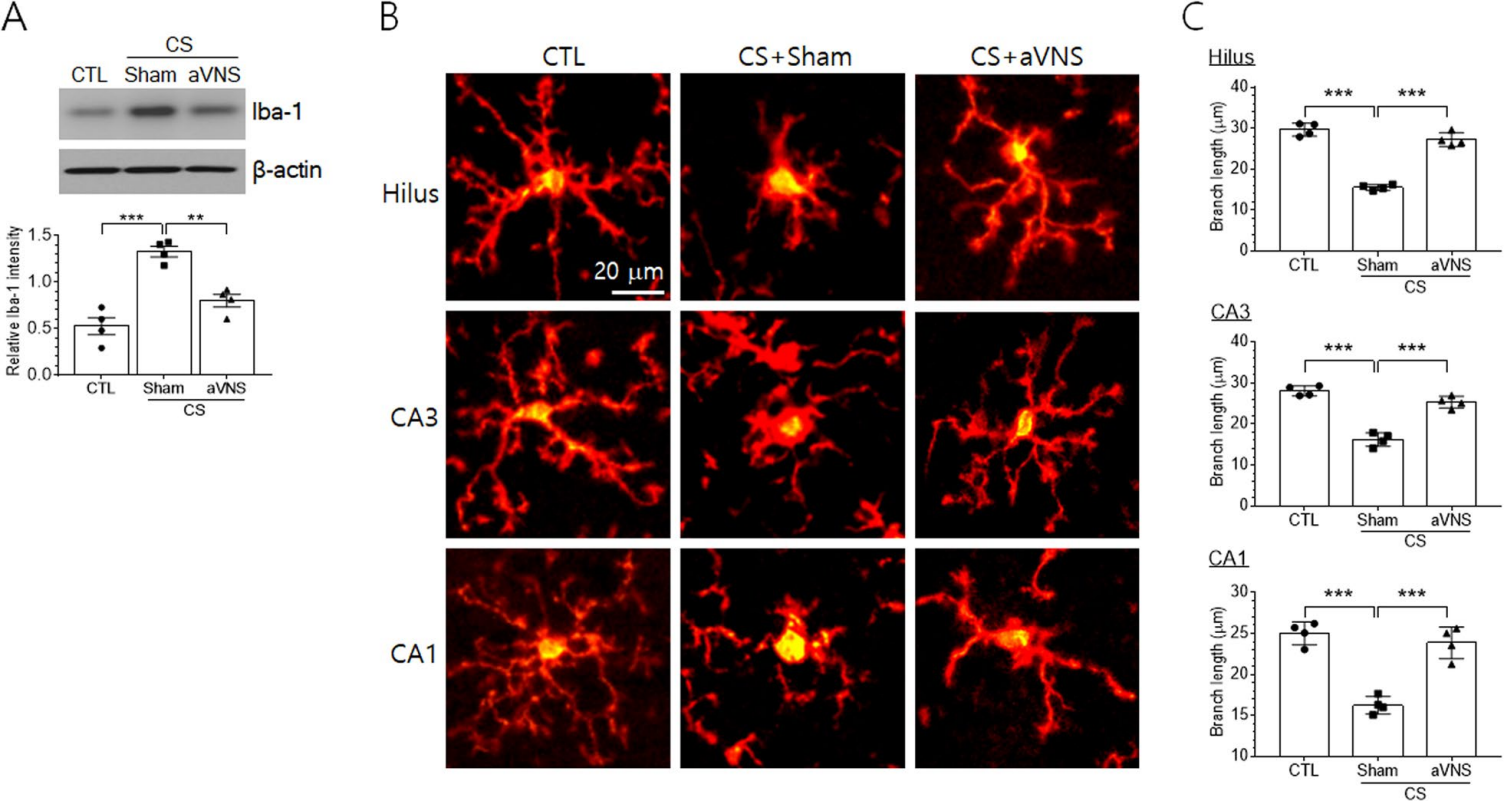
Microglial activation in the hippocampus of CS animals is controlled by aVNS. **A** Western blot analysis of Iba-1 protein. Upper images are the representatives from four independent experiments. Quantification of protein band intensity relative to actin is plotted (lower panel). **B** Immunofluorescence view of Iba-1-labeled microglial cells. For three experimental groups, representative cell images were taken from the hilus region and the striatum radiatum areas of CA3 and CA1 pyramidal cells. **C** Quantification of branch length of microglial cells. Branch length was averaged by measuring randomly selected 15–20 cells per animal and the data from 4 independent animal preparations were compared among experimental groups. **p < 0.01, ***p < 0.001 (One-way ANOVA)

To investigate whether the regulation of inflammatory cytokine production involved the activation of microglial cells, we analyzed Iba-1, a marker protein of microglial cells in the hippocampus. Similar to inflammatory cytokines, Iba-1 protein level was elevated in the hippocampus of CS animals and downregulated by aVNS (Fig. 3A). Immunofluorescence labeling of microglial cells with Iba-1 showed that the cell morphology was highly branched and elongated in hippocampal subfields such as hilus region and dendritic zones of CA3 and CA1 pyramidal cells in the control animals and changed to a less ramified shape with expanded cell body in CS animals (Fig. 3B). In CS+VNS group, the microglial processes were clearly extended, showing a similar morphology as in the control group (Fig. 3B). Quantitative assessment of the mean length of microglial branch showed significant decreases in all three hippocampal subfields of CS group animals and increases by VNS to similar levels as those in the control group (Fig. 3C).

### Cholinergic synaptic transmissions are augmented by aVNS in the hippocampus

**Fig. 4:**
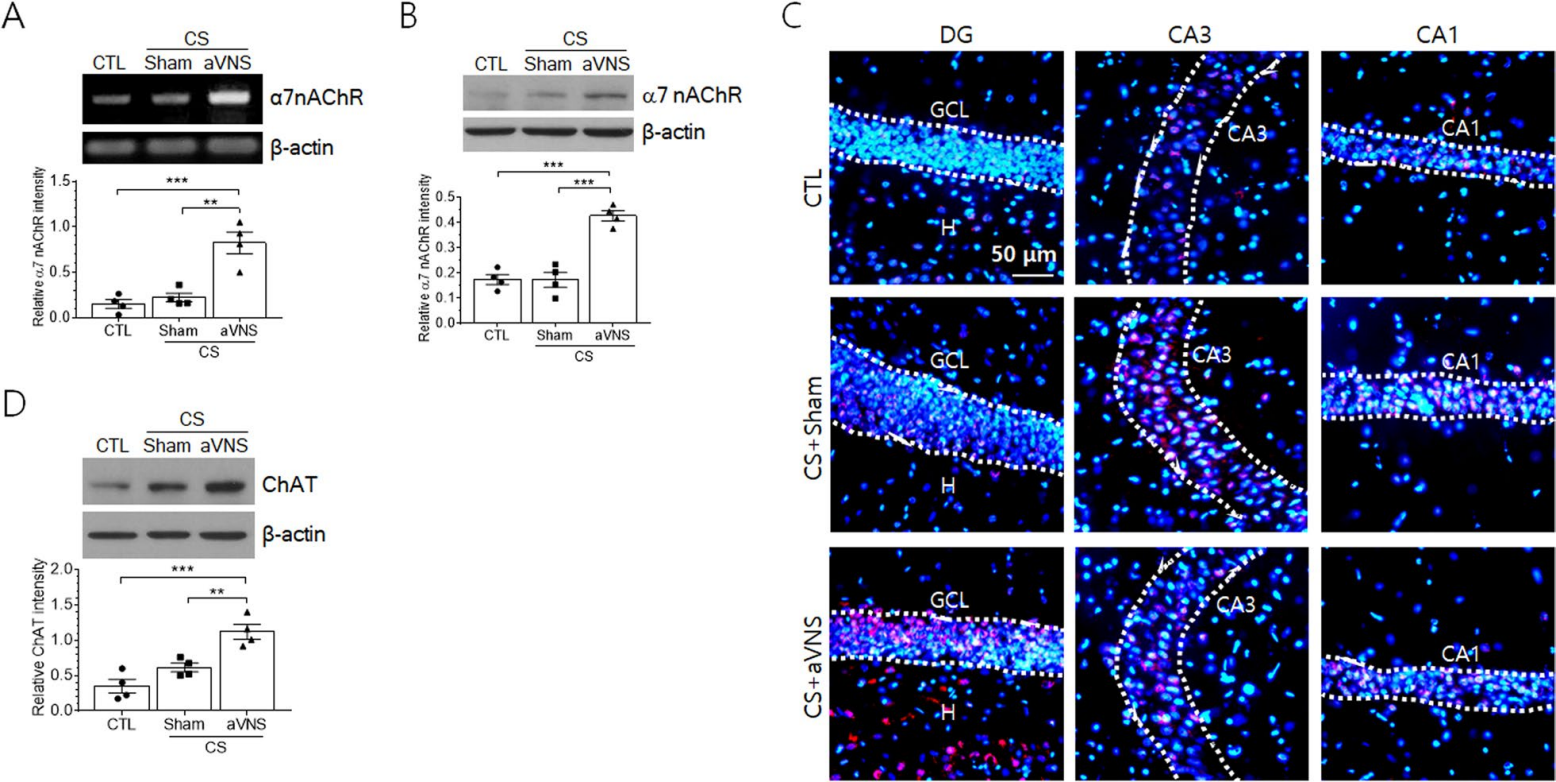
aVNS upregulates the expression of α7 nAChR and ChAT in the hippocampus of CS animals. **A** RT-PCR analysis of α7 nAChR mRNA expression in the hippocampus. Upper images of agarose gel electrophoresis are the representatives of four independent experiments. Quantification of DNA band intensity relative to actin is plotted in lower panel. **B** Western blot analysis of α7 nAChR in the hippocampal tissues. **C** Immunofluorescence images of α7 nAChR signals in the granule cell layer (GCL) of the dentate gyrus (DG) and CA3 and CA1 pyramidal cell layers. The nuclei of neurons were visualized by staining with Hoechst 33258 (blue). **D** Western blot analysis of ChAT protein in the hippocampal tissue. In **B** and **D**, upper images are the representatives from four independent experiments. Quantification of band intensity relative to actin is plotted (lower panel). **p < 0.01, ***p < 0.001 (One-way ANOVA)

While cholinergic anti-inflammatory pathway via the activation of α7 nAChR has been well demonstrated in several visceral organs of experimental animals given VNS, its effects in the central nervous system remain largely unknown. Given that α7 nAChR is expressed in several brain tissues including hippocampus and innervated by the projection of cholinergic afferents from the septo-diagonal band of Broca [26–28], here we investigated possible involvement of α7 nAChR in the process of VNS-mediated regulation on the hippocampal production of inflammatory cytokines. Levels of hippocampal α7 nAChR mRNA were not changed by CS but significantly increased by VNS (Fig. 4A). Likewise, α7 nAChR protein was similarly regulated by VNS, showing no change by CS and elevation by VNS (Fig. 4B). α7 nAChR signals were detected in CA3 and CA1 pyramidal cell layers in control and CS groups of animals. α7 nAChR signals were induced clearly in the granule cell layer of CS+VNS group (Fig. 4C). It was also noted that the protein signals were detected in the hilus region of CS+VNS group of animals. To further investigate whether VNS affected cholinergic inputs into the hippocampus, we examined the production of cholinergic acetyltransferase (ChAT) in the hippocampus. ChAT was expressed at moderate level in the hippocampus of control group and slightly elevated in CS group without showing statistical significance (*p* = 0.15, One-way ANOVA). ChAT level was further increased by VNS compared to the control or CS group of animals (Fig. 4D).

**Fig. 5:**
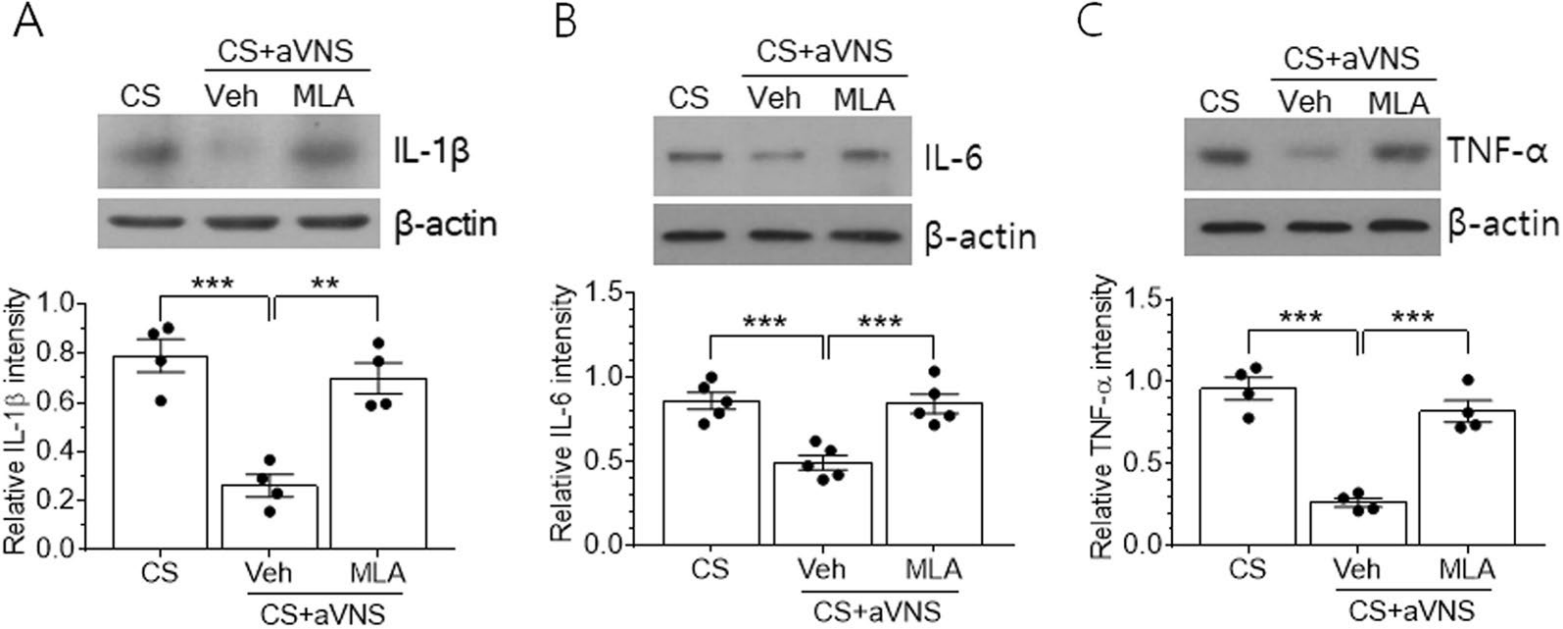
Effects of α7 nAChR inhibitors on the hippocampal production of inflammatory cytokines. An α7 nAChR inhibitor MLA or saline vehicle solution (Veh) was administered 20 min prior to aVNS in CS animals, and proteins were extracted from hippocampal tissues for western blot analysis. Upper images in **A**–**C** are the representatives of 4–5 independent experiments for IL-1β, IL-6, and TNF-α proteins. Quantification of protein band intensity relative to actin is plotted in lower panels. **p < 0.01, ***p < 0.001 (One-way ANOVA)

To further examine whether the activity of α7 nAChR is required for VNS-mediated anti-inflammation, we analyzed inflammatory cytokine levels after the intraventricular injection of MLA, an α7 nAChR antagonist. Levels of IL-1β, IL-6, and TNF-α, which had been markedly decreased by VNS in CS animals, were significantly elevated by MLA treatment, leading to similar levels as those of the corresponding CS group of animals in all three proteins (*p* = 0.54, 0.54, and 0.97 for IL-1β, IL-6, and TNF-α, respectively) (Fig. 5A–C).

### Regulation of cell death and serotonergic receptor production by chronic VNS

**Fig. 6:**
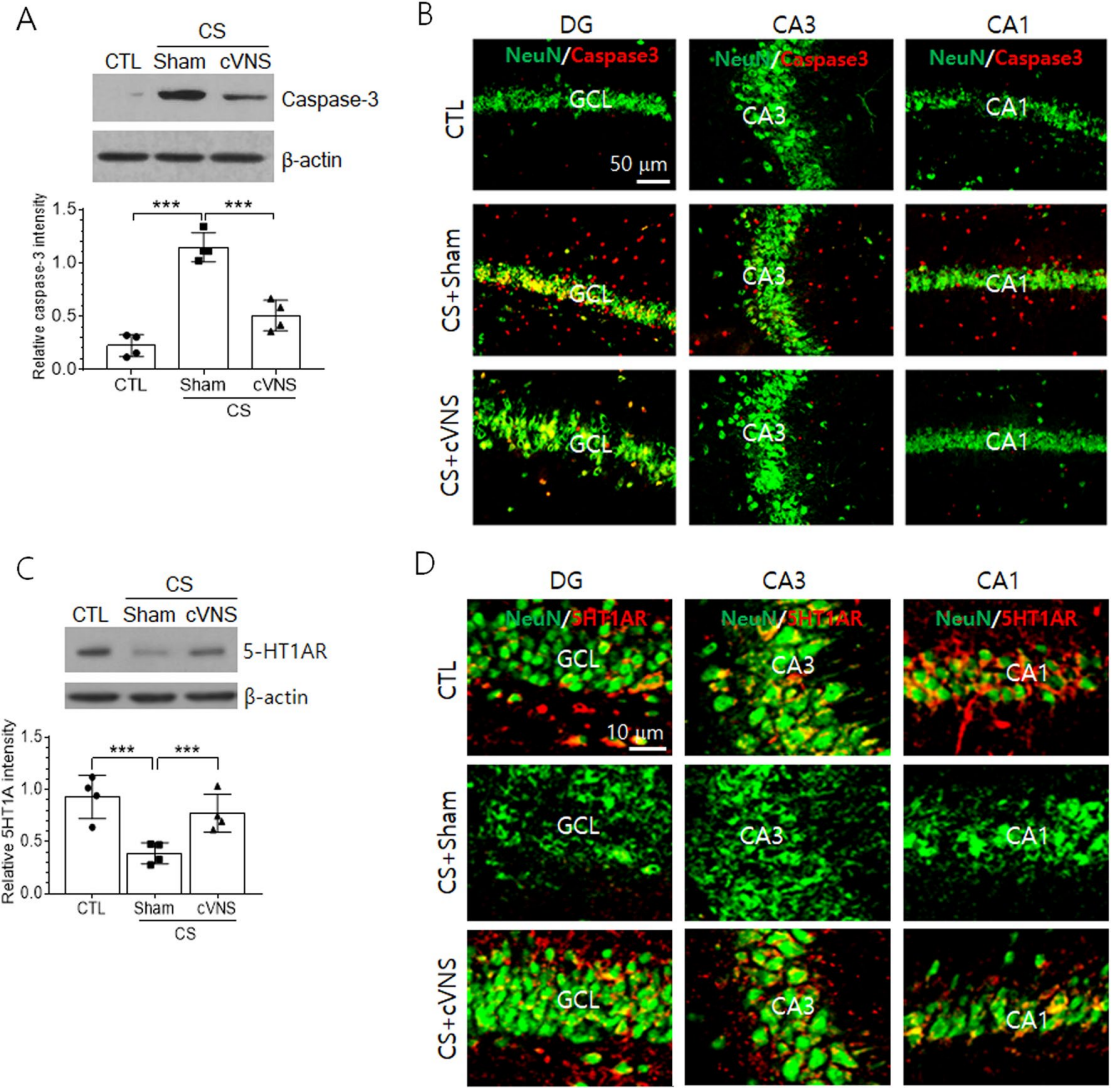
Effects of cVNS on the production of active form of caspase 3 and 5-HT1A receptors in the hippocampus of CS animals. **A**, **C** Western blot analysis of cleaved active form of caspase 3 (**A**) and 5-HT1A receptors (**C**) in the hippocampus. Images in **A** and **C** are the representatives from four independent experiments. Quantification of band intensity relative to β-actin is plotted in lower panels. ***p < 0.001 (One-way ANOVA). **B**, **D** Immunofluorescence staining of active caspase 3 (red in **B**) and 5-HT1AR (red in **D**) merged with NeuN (green) in the granule cell layer (GCL) of dentate gyrus (DG) and CA3 and CA1 pyramidal cell layers

To determine whether the chronic application of VNS affects hippocampal neuronal activity and behavioral consequences in CS animals, we implanted the microelectrode and stimulated the vagus nerve for 7 days. Cleaved, active form of caspase 3 was very low in the intact hippocampal tissue and was strongly induced by CS. Then, the chronic application of VNS resulted in significant reduction of caspase 3 levels (Fig. 6A). Immunofluorescence staining showed that cleaved caspase 3 signals, which were induced from the granule cells in the dentate gyrus and CA3 and CA1 pyramidal cells of CS animals as identified by colocalization with NeuN, were attenuated by chronic VNS (Fig. 6B). Cleaved caspase 3-positive cells that were detected from the outside of neuronal cell layers in CS group were also downregulated in CS+VNS group. We further investigated the effects of cVNS on the production of 5-HT1AR in hippocampal neurons. Moderate level of 5-HT1AR protein was detected in the hippocampal tissue of the control group and the protein level was significantly reduced in CS group (Fig. 6C). Then, the application of cVNS to CS animal significantly elevated 5-HT1AR level in the hippocampus. Immunofluorescence localization clearly showed the presence of 5-HT1AR signals in neurons of granule cell layer and CA1 pyramidal cell layers in the control group (Fig. 6D). Reduction and re-induction of 5-HR1AR signals were clearly observed in the granule cell layer and CA3 and CA1 pyramidal cell layers in CS and CS+VNS groups of animals, respectively.

### cVNS is involved in regulating pain sensitivity and depressive-like behavior

**Fig. 7:**
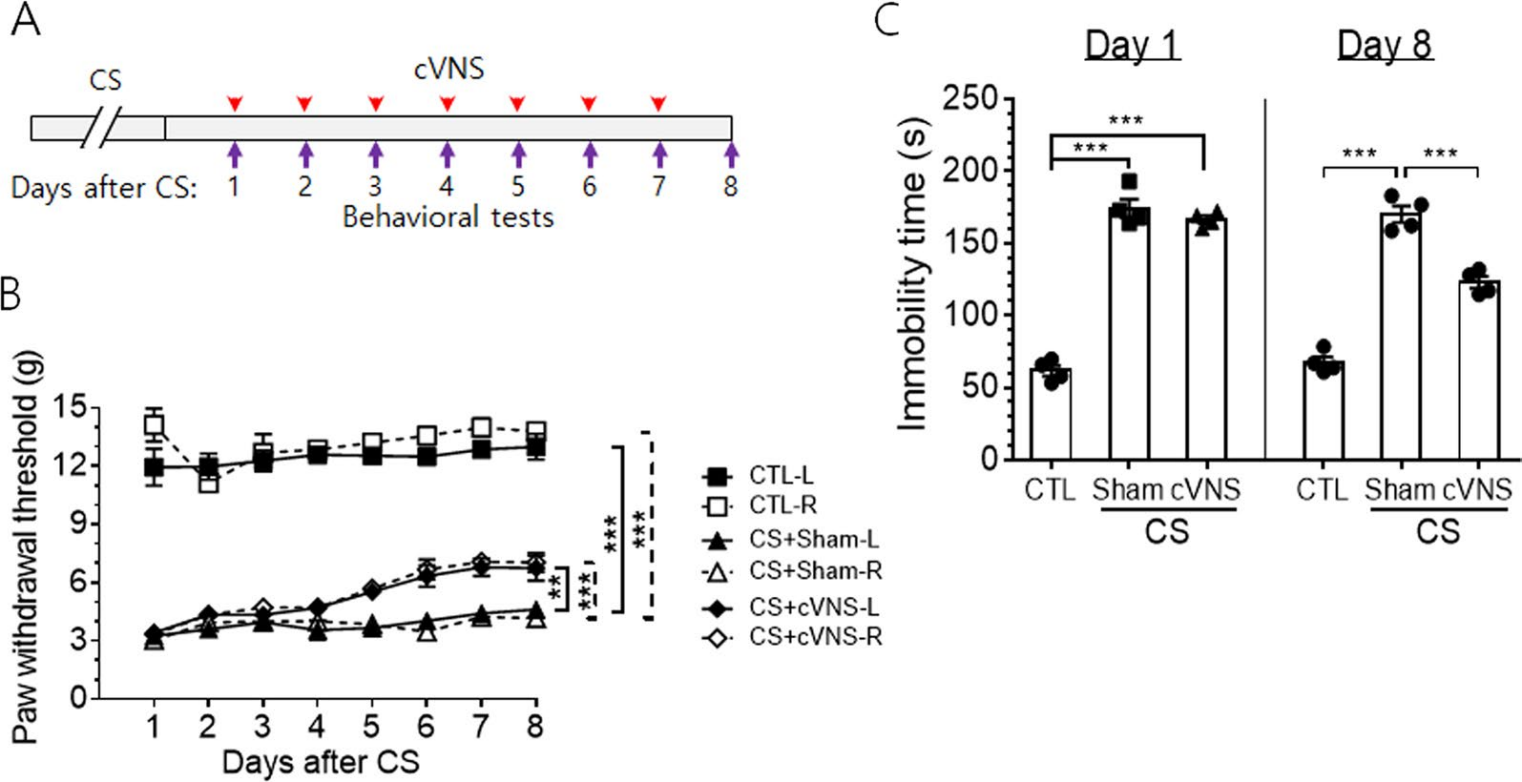
cVNS improves pain response and depressive-like behavior in CS animals. **A** A schematic diagram showing animal treatments including behavioral tests. VNS was initiated 24 h after final CS and given for 7 consecutive days, Behavioral tests were performed immediately after cVNS and continued each day for 8 days. **B** Von Frey hair test. Values of withdrawal threshold to stimulations on the left (L) and right (R) hind limbs were measured 20 times for individual animals as indicated in the Figure. Statistical comparison among groups were made by two-way ANOVA repeated measures (**p < 0.01, ***p < 0.001; see ‘Results’ section for details). In the plot in (**B**), vertical box brackets with solid and dashed lines denote the comparisons between the corresponding solid symbols and between the open symbols, respectively. **C** Forced swimming test. Immobility time was measured on the first and the eight days after CS (labeled day 1 and day 8 in the figure). ***p < 0.001 (One-way ANOVA, *n* = 4)

To examine the effects of cVNS on animal behaviors, we analyzed pain sensitivity in the hind limb by von Frey test and immobility response as an indicator of depressive-like behavior by forced swimming test on a daily basis for 8 days after CS (vertical arrows in Fig. 7A). Pain responses were similar between the left and right hind limbs in each experimental groups (*p* = 0.2 for CTL-L and CTL-R, *p* > 0.99 for CS-sham-L and CS-sham-R, *p* = 0.99 for CS-VNS-L and CS-VNS-R; Two-way ANOVA, *df* = 1, *n* = 4). Pain sensitivity, as measured by paw withdrawal threshold, was significantly increased in CS animals compared to the control group and remained at the similar level during the period of VNS application (CTL-L vs. CS+Sham-L; *p* < 0.0001, CTL-R vs. CS+Sham-R; *p* < 0.0001, two-way ANOVA *df* = 1, *n* = 4; Fig. 7B). Pain sensitivities were then gradually recovered 5 days after applying chronic VNS (CS+Sham-L vs. CS+VNS-L; *p* = 0.0022, CS+Sham-R vs. CS+VNS-R; *p* = 0.0005, two-way ANOVA *df* = 1, *n* = 4). In the forced swimming test, immobility time duration was greatly increased in CS group of animals and maintained the similar level when measured 1 day after the last CS with a single bout of VNS (left plot in Fig. 7C). Immobility time remained elevated 7 days after CS, but the score was significantly decreased in CS+cVNS animals after 7 bouts of VNS when analyzed 8 days after CS (right plot in Fig. 7C).

### Behavioral effects of cVNS on CS animals are linked to gastric and hippocampal inflammations

**Fig. 8:**
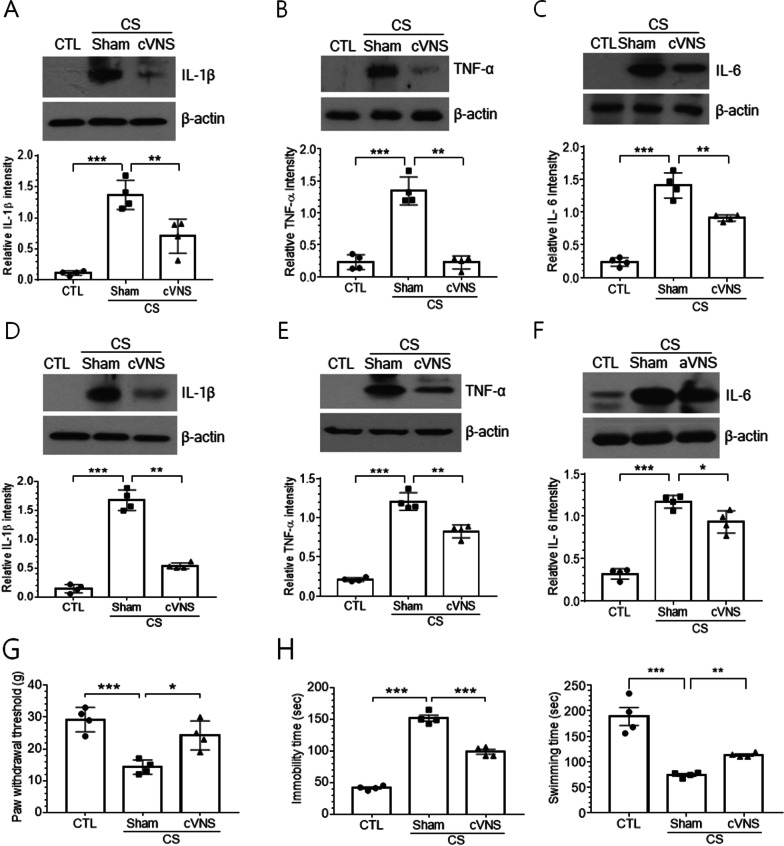
Regulation of gastric and hippocampal inflammation by cVNS is related to behavioral effects. Rats were subjected to CS (5 days) and subsequently cVNS (7 days), and 24 h after the last VNS, CS+cVNS group, along with CS+Sham and CTL groups, were used for von Frey test (**G**) and forced swimming test (**H**). Animals were then sacrificed and protein was extracted from the gastric tissue (**A**–**C**) and the hippocampus (**D**–**F**) for Western blot analysis. **A**–**F** Western blot analysis. Upper images are the representatives of four independent experiments for IL-1β, TNF-α and IL-6 proteins in the gastric tissue (**A**–**C**) and in the hippocampal tissue (**D**–**F**). Quantification of protein band intensity relative to actin is plotted in lower panels. *p < 0.05, **p < 0.01, ***p < 0.001 (One-way ANOVA). **G** Von Frey hair test. Values of withdrawal threshold to stimulations on the left hind limbs were measured 40 times and averaged for individual animals. **H** Forced swimming test. Duration of immobility and swimming were analyzed for 5 min period for individual animals. In **G** and **H**, mean behavioral data collected from four different animals were compared among experimental groups. *p < 0.05, **p < 0.01, ***p < 0.001 (One-way ANOVA)

To further examine whether behavioral improvements by cVNS were related to the regulation of inflammatory responses, we analyzed levels of IL-1β, TNF-α and IL-6 in the gastric and hippocampal tissues. Gastric levels of these inflammatory cytokines were strongly induced in CS+Sham group of animals and then significantly decreased by cVNS (Fig. 8A–C). Similarly, IL-1β, TNF-α and IL-6 protein levels in the hippocampus were highly induced by CS and significantly decreased by cVNS (Fig. 8D–F). To examine whether the cVNS-mediated regulation of inflammation is related to animals’ behavior, we performed behavioral tests for the same set of animals that were subsequently subjected to the investigation of gastric and hippocampal inflammation. Pain sensitivity as measured by paw withdrawal threshold was greatly increased by CS and reduced by cVNS (Fig. 8G). In the forced swimming test, CS-induced increase of immobility time was significantly reduced by cVNS, whereas the swimming time was significantly improved by cVNS (Fig. 8H).

**Fig. 9:**
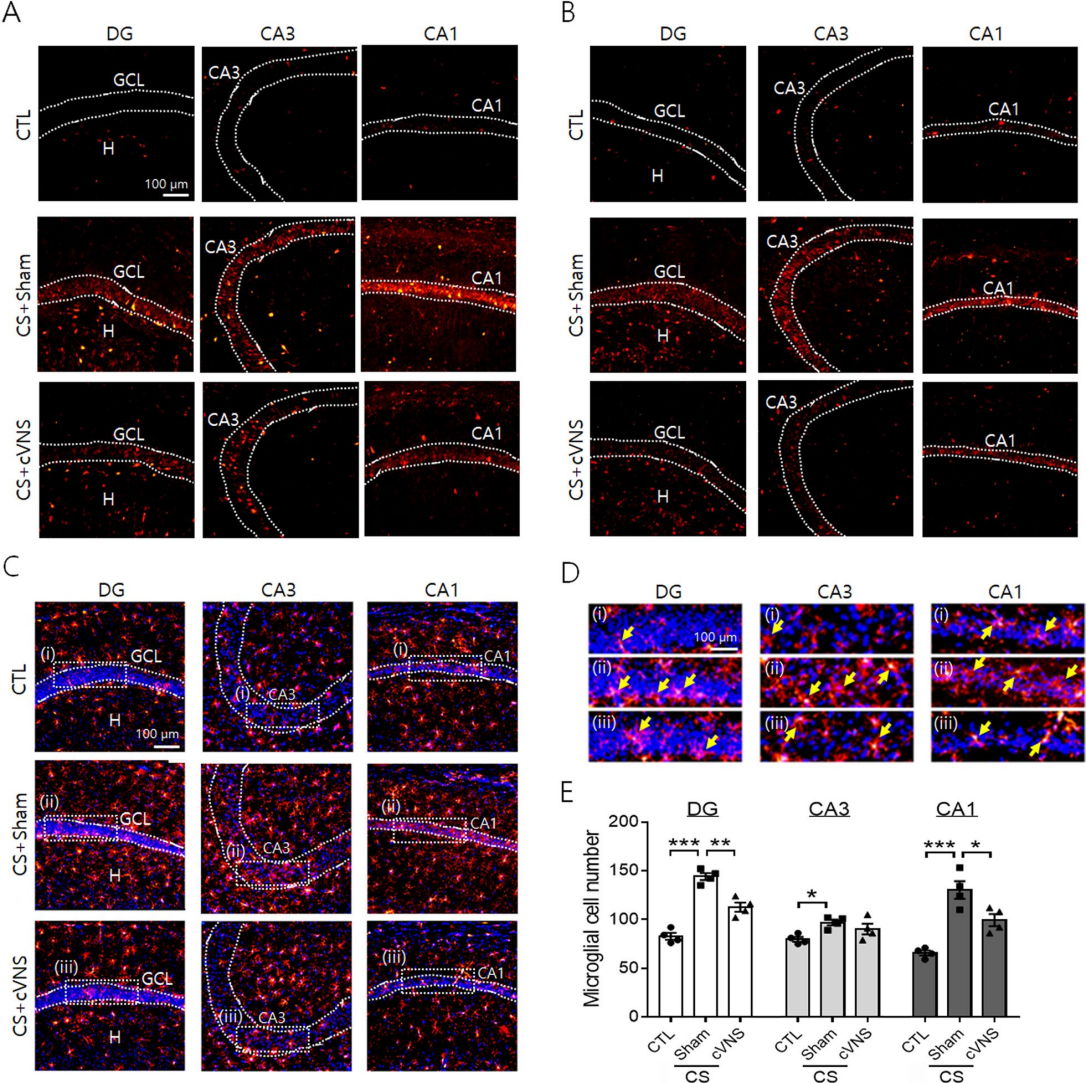
Immunofluorescence localization of IL-1β, TNF-α and Iba-1 proteins in hippocampal subfields after cVNS in CS animals. In **A** and **B** images showing IL-1β and TNF-α protein signals (in red), respectively, borders of granule cell and CA3 and CA1 pyramidal cell layers were demarcated after merging with Hoechst nuclear-stained images (not shown). **C**, **D** Immunofluorescence view of Iba-1-labeled microglial cells (red) in the hippocampus. Hoechst-stained nuclei demarcated the neuronal cell layers (dotted lines). Images in **D** are the enlarged view of rectangle areas in (**C**). Yellow arrows indicate Iba-1-labeled microglial cells in the granule cell layer (GCL) in dentate gyrus (DG) and CA3 and CA1 pyramidal regions. **E** Quantification for the number of the microglial cells in the microscopic fields (4.3 × 4.3 µm^2^) in the areas of dentate gyrus (DG) and CA3 and CA1 hippocampal formation. Cell numbers in the microscopic fields were averaged by counting 8 to 10 nonconsecutive sections per hippocampal tissue and the mean data from 4 hippocampal preparations were compared among experimental groups. 0.05, **p < 0.01, ***p < 0.001 (One-way ANOVA)

We further examined the production of inflammatory cytokines in the hippocampal subfields by immunofluorescence staining. Both IL-1β and TNF-α proteins were highly induced by CS in granule cells in the dentate gyrus and CA3 and CA1 pyramidal cells, and the signal intensity was decreased by cVNS (Fig. 9A, B). Immunofluorescence-labeling of microglial cells also revealed clear increases in cell number in the hippocampal regions of dentate gyrus and CA3 and CA1 subfields after CS (Fig. 9C, E). In CS animals, Iba-1-labeled cells were frequently found within or around the border of hippocampal cell layers. For instance, microglial cells were detected in the inner part of the granule cell layer, and they were also seen within the CA3 and CA1 pyramidal cell layers (yellow arrows in Fig. 9D). The density of Iba-1-labeled microglial cells was reduced in the subfields of dentate gyrus and CA1, but not in CA3 area (Fig. 9E). Microglial cells were found less in the granule cell layer and CA1 pyramidal cell layer in CS+cVNS compared to CS+Sham group (Fig. 9D).

## Discussion

Previous studies have reported that microglial cells are activated in the spinal cord of CS animals and related to chronic muscle pain [2]. Here, we provide evidence that CS increases the production of inflammatory cytokines in the hippocampus and an application of VNS either in an acute or a chronic form attenuates inflammation and improves pain response and depression-like behavior in CS animals. Pharmacological blockade of α7 nAChR abrogated the suppressive effects of VNS on the production of inflammatory cytokines, suggesting the requirement of cholinergic nerve activity for anti-inflammation.

A multiple continuous stress model was originally developed to study gastric ulcer, ischemia, and dysfunction in gastric motility [29, 30]. In addition, CS animals show alterations in endocrine function and chronic pain [4, 31]. Having noted that abnormal regulation of gastrointestinal function in CS animals can affect brain function and the vagus nerve acts as a bridge connecting between gut and brain (brain–gut axis) [7], we explored the potential regulatory function of vagus nerve activity in CS-related neuroinflammation and neurological abnormalities. We found that CS consistently increased levels of TNF-α, IL-1β, and IL-6 in the gastric and hippocampal tissues and induced morphological changes of microglial cells. Also, the number of microglial cells were increased in the hippocampal subfields including granule cell layer and CA3 and CA1 pyramidal cell layers, suggesting CS-mediated induction of the proliferation and migration activities of microglial cells. In several neurodegenerative diseases such as Alzheimer’s disease, Parkinson’s disease, amyotrophic lateral sclerosis (ALS), and prion disease, neuroinflammation in brain tissues is noted with dystrophic morphology of microglial cells [32, 33], and here the microglial cells produce reactive oxygen species (ROS), phagocytic receptors, proinflammatory cytokines and others causing neurotoxic effects [34]. It is thus presumed that microglial cell may interact with neurons and contribute to neuroinflammation in CS animals.

The vagus nerve is composed of afferent and efferent components of which 80% account afferent fibers [35], implicating the significance of afferent vagus signaling in mediating brain–gut axis. Afferent vagus nerve activity is transmitted to raphe nucleus, brachial nucleus, locus coeruleus and ventral tegmental nucleus and further propagated into the forebrain areas [7, 16, 36]. Stimulation of visceral sensory fibers of the vagus nerve activates brain’s reward system [16]. Moreover, preclinical and animal studies provide evidence that VNS can improve the symptoms of epilepsy, depression, and tinnitus [13, 14, 21, 37, 38], and also regulate neuroinflammation in the brain of animals injected with 6-OHDA and in LPS-injected animals for endotoxemia [39–41].

Here, we found that both acute and chronic forms of VNS downregulated the production of IL-1β, TNF-α and IL-6 in the hippocampus of CS animals and the cholinergic nerve activity was involved in VNS-modulated neuroinflammation. Cholinergic nerve fibers are projected into the hippocampus mainly through the medial septum-diagonal band of Broca and activate cholinergic receptors in target cells in the hippocampus [26–28]. Our data show that α7 nAChR proteins are expressed at moderate levels in the hippocampus of both control and CS animals and significantly increased by VNS. While our data showing the increased production of inflammatory cytokines by α7 nAChR antagonist MLA in CS+VNS animals indicate the requirement of α7 nAChR activity for VNS-mediated anti-inflammation, it is unknown at this moment how VNS-mediated activation of α7 nAChR is linked to the signaling pathway leading to decreased expression of inflammatory cytokines in hippocampal neurons. According to our data, inflammatory cytokine IL-1β was mostly induced from hippocampal neurons of CS animals and downregulated by VNS. It was reported that the activation of α7 nAChR in microglial cells was related to LPS-induced transition of microglial cell M1 and M2 [42]. Augmented cholinergic inputs could affect directly on microglial cells or indirectly through the postsynaptic neurons. Neuron–microglial communication is made through chemical mediators such as inflammatory cytokines, metalloproteases, and chemokine signaling and plays a determining role in neuronal survival [33, 43, 44]. It will be of great interest to determine whether VNS modulates the effects of fractalkine–CX3CR1 signaling for the regulation of microglial neurotoxicity in the hippocampus of CS animals [45]. Finally, VNS increased levels of ChAT enzymes in the hippocampus, implying the increased production of acetylcholine in the presynaptic terminal and possibly contributing to presynaptic facilitation. Coincidental activation for the synthesis of presynaptic neurotransmitters and the postsynaptic receptors may facilitate the anti-inflammatory activity of VNS in target neurons in the hippocampus.

ME/CFS, representing the diverse abnormal pathophysiological processes in gastrointestinal, immune, and psychobiological functions, with high commodities, does not reveal clear etiology so far. One of the pathophysiological features of ME/CFS is a decrease in parasympathetic activity, showing reduced activation of α7 AChR [46]. It was also reported that the activation of α7 nAChR in microglial cells increases mitochondrial activity which is related to neuroprotection [47, 48]. This notion is consistent with our current data showing the role of VNS-induced α7 nAChR activity in regulating neuroinflammation.

## Conclusion

This study demonstrates that VNS attenuates hippocampal neuroinflammation caused by CS in rats and improves pain sensitivity and depressive-like behavior. Given that CS animals partially show the symptoms associated with CFS [2], VNS may be considered as the possible therapeutic strategy for CFS known to involve complex neuroimmune activities, as neuroimmune communication is a key principle explaining VNS efficacy in the brain (e.g., neuron–microglial interaction) as well as in the visceral organs [49].

## Data Availability

All data generated and analyzed during this study are available from the corresponding author on reasonable request.

## Abbreviations

CFS: Chronic fatigue syndrome
VNS: Vagus nerve stimulation
aVNS: Acute vagus nerve stimulation
cVNS: Chronic vagus nerve stimulation
CS: Continuous stress
α7nAChR: α7 nicotinic acetylcholine receptor
5-HT1AR: 5-HT1A receptor
ChAT: Cholinergic acetyltransferase
DG: Dentate gyrus
GCL: Granule cell layer
