# Correction to: Vagus nerve stimulation modulates hippocampal inflammation caused by continuous stress in rats

**DOI:** 10.1186/s12974-022-02536-5

**Published:** 2022-07-06

**Authors:** Uk Namgung, Ki-Joong Kim, Byung-Gon Jo, Jong-Min Park

**Affiliations:** Department of Oriental Medicine, Institute of Bioscience and Integrative Medicine, Daejeon University, Daehak-ro 62, Daejeon, 34520 South Korea

## Correction to: Journal of Neuroinflammation 19:33 (2022) https://doi.org/10.1186/s12974-022-02396-z

Following publication of the original article [1], the authors identified that few corrections were missed out and not carried out properly. It has been corrected in this correction.

1. The author Dr. Jong-Min Park’s name should be displayed as ‘Jong-Min Park’
2. Ref [28] is published incompletely. The complete reference is given below:
  Lawrence JJ, Cobb S. Neuromodulation of hippocampal cells and circuits. In: Cutsuridis V, Graham BP, Cobb S, Vida I, editors. Hippocampal microcircuits: a computational modeler’s resource book. 2nd ed. Cham: Springer; 2018. pp. 227–325.
3. In “Background” section, the word “Schizophrenia” in the paragraph 2, left column line number 17 should be “schizophrenia”.
4. In “Results” section, under the heading “cVNS is involved in regulating pain sensitivity and depressive‑like behavior” left column line 12: “each experimental groups” should read as “each experimental group”.
5. In Fig. 7 legend, last line: “the eight days” should read as “the eighth days”
6. In Fig. 9 legend, second line from the bottom: *p was missing in the value 0.05. It should read as “*p < 0.05”

The original article [1] has been corrected.
